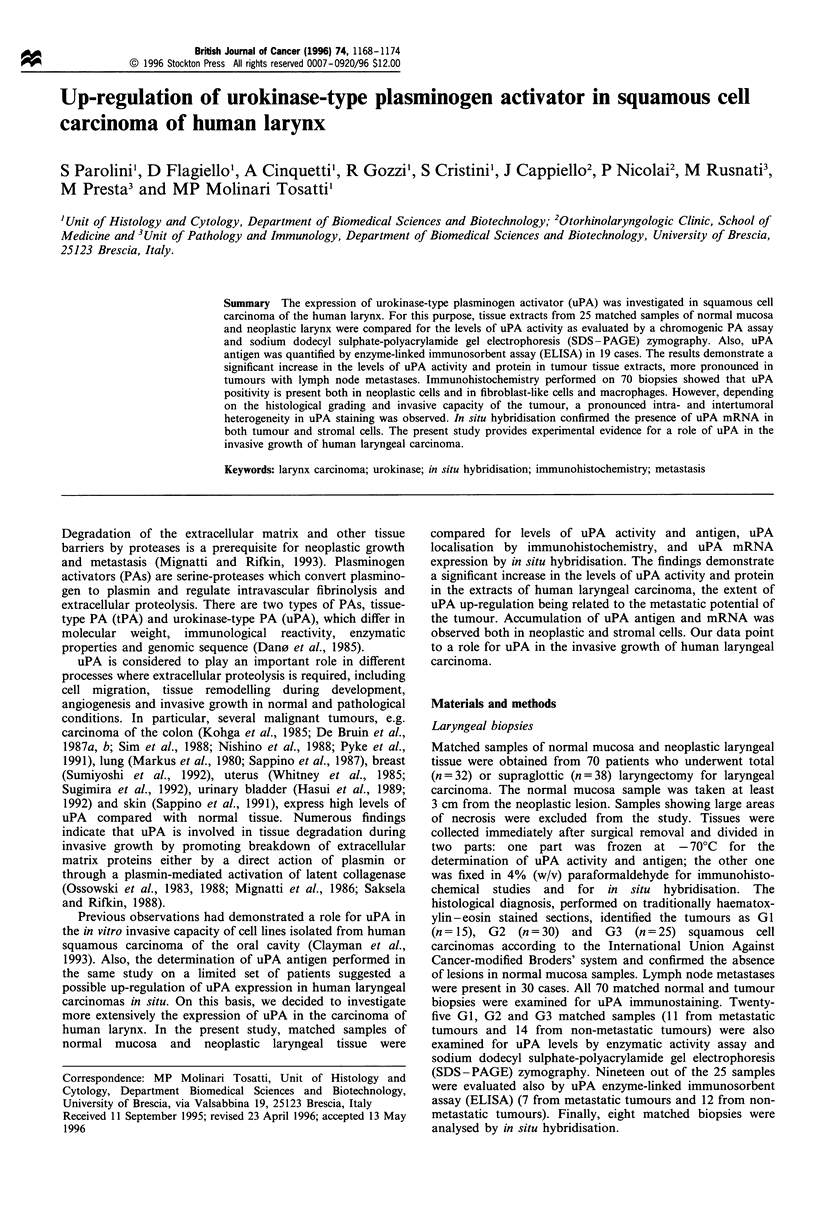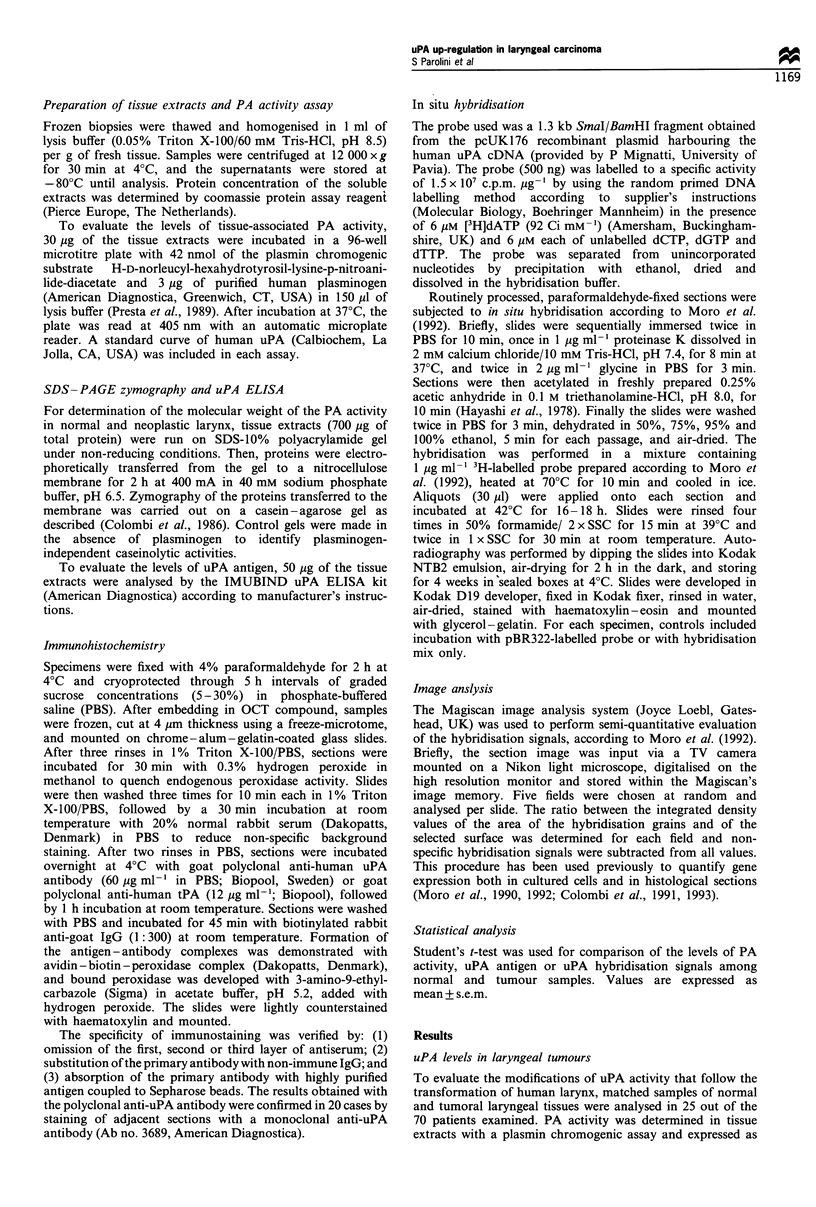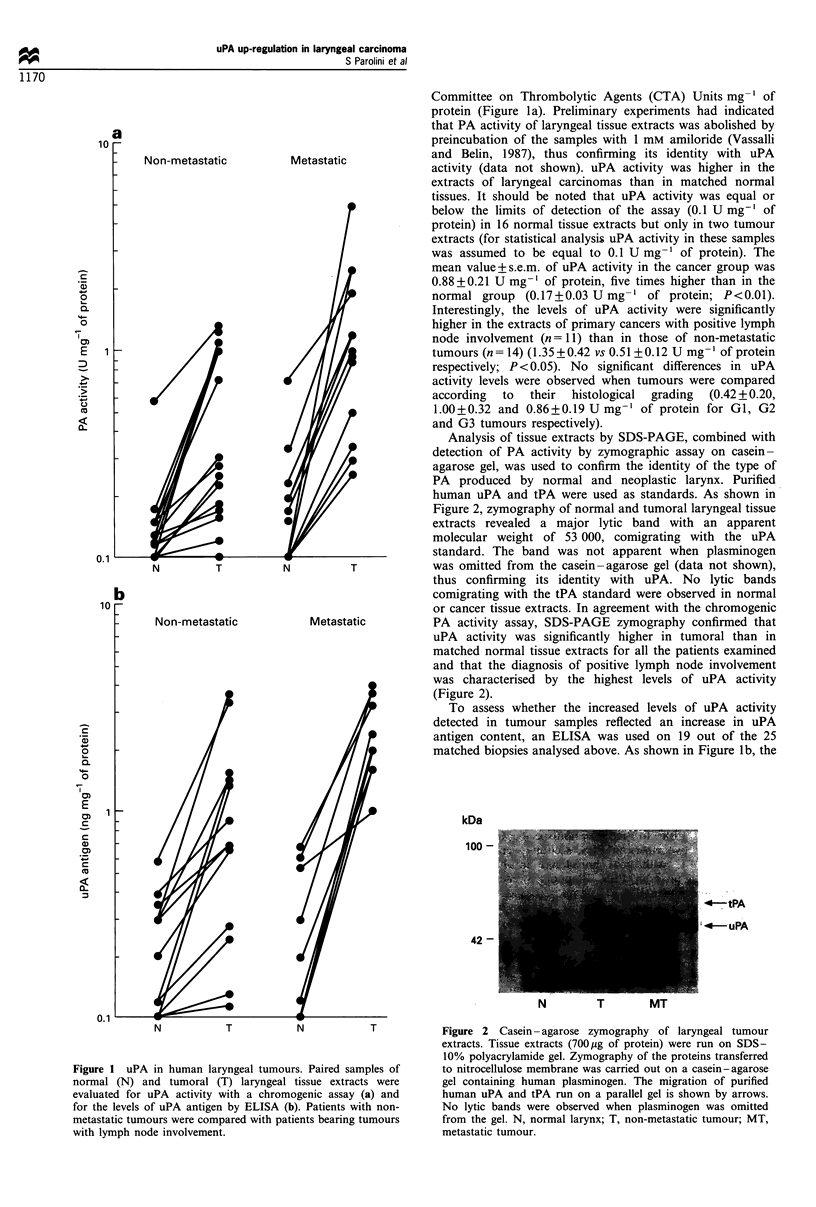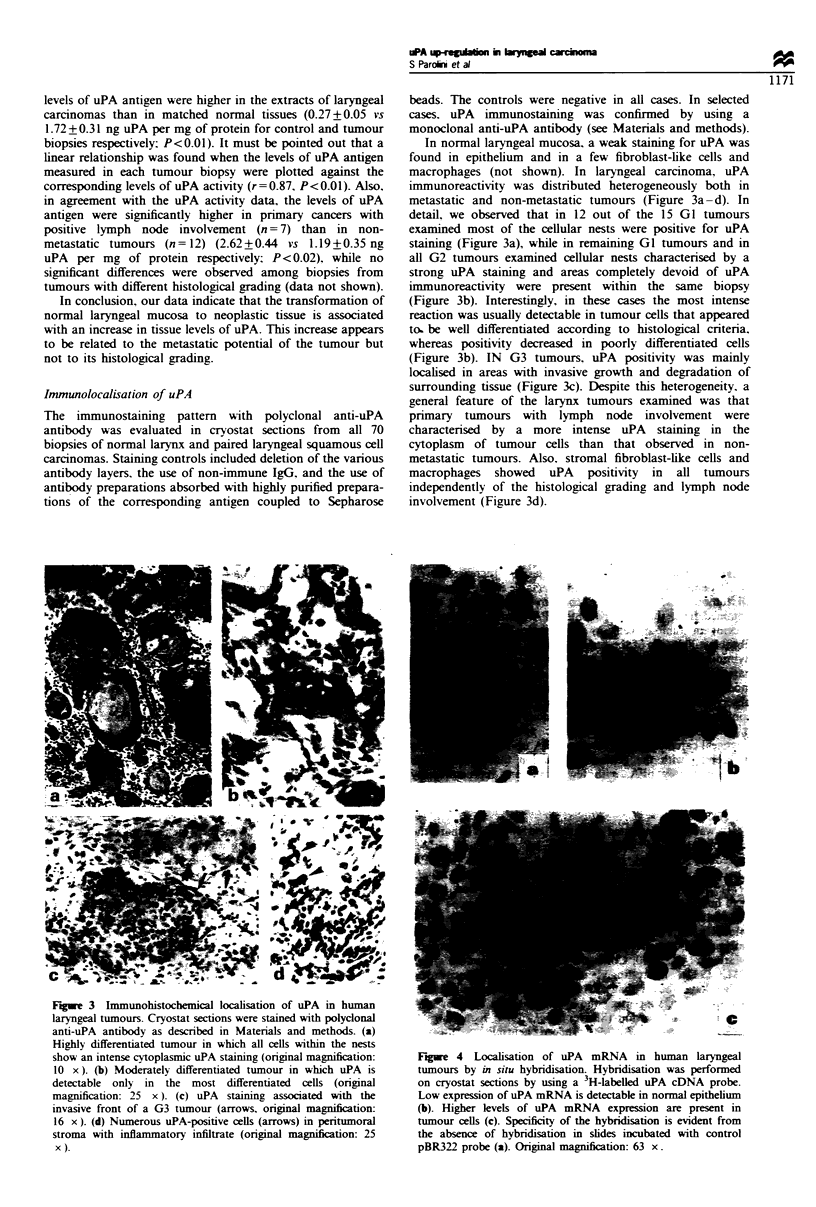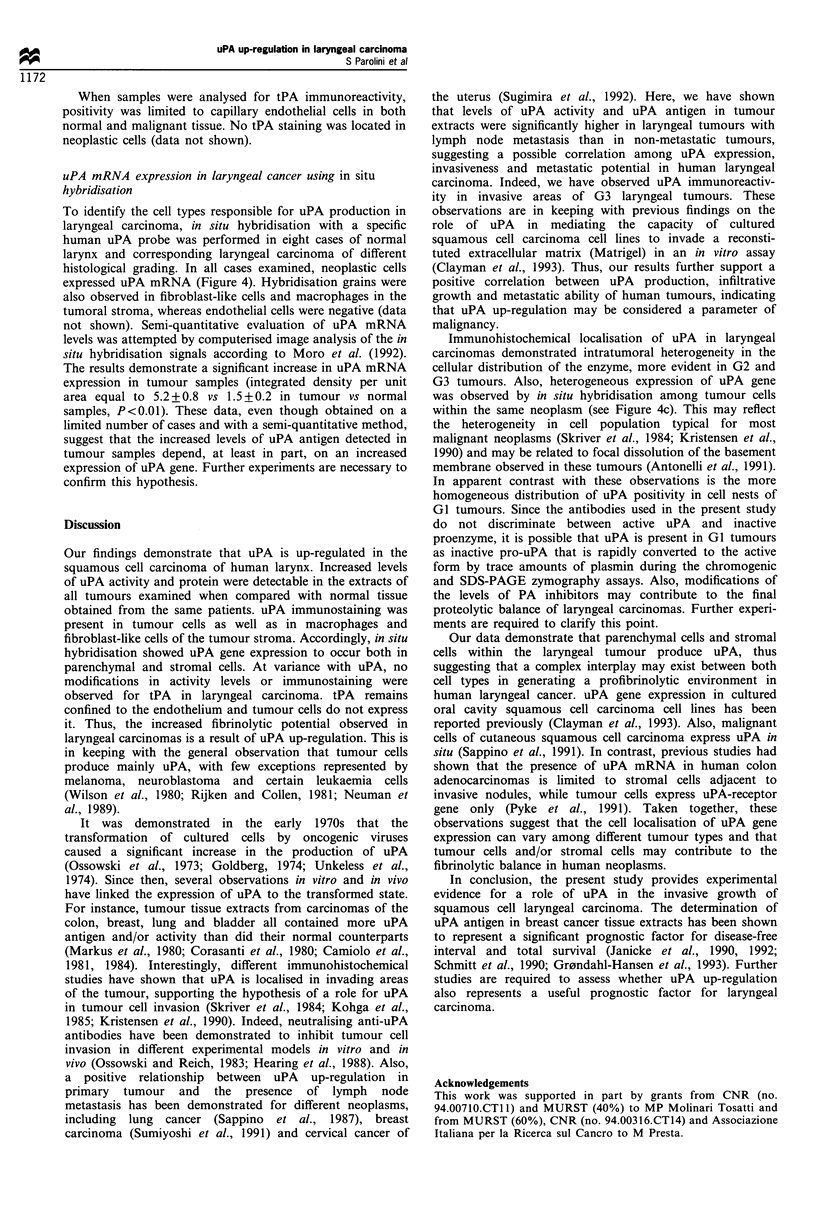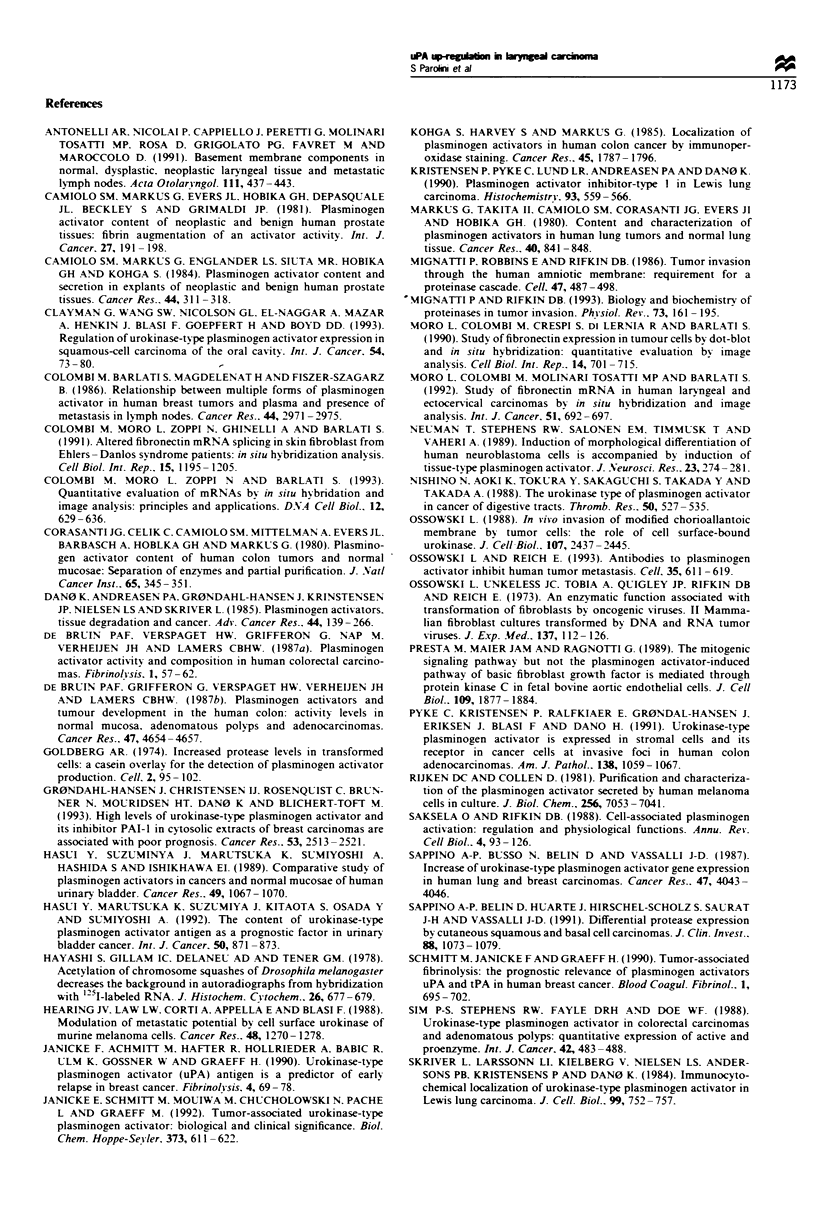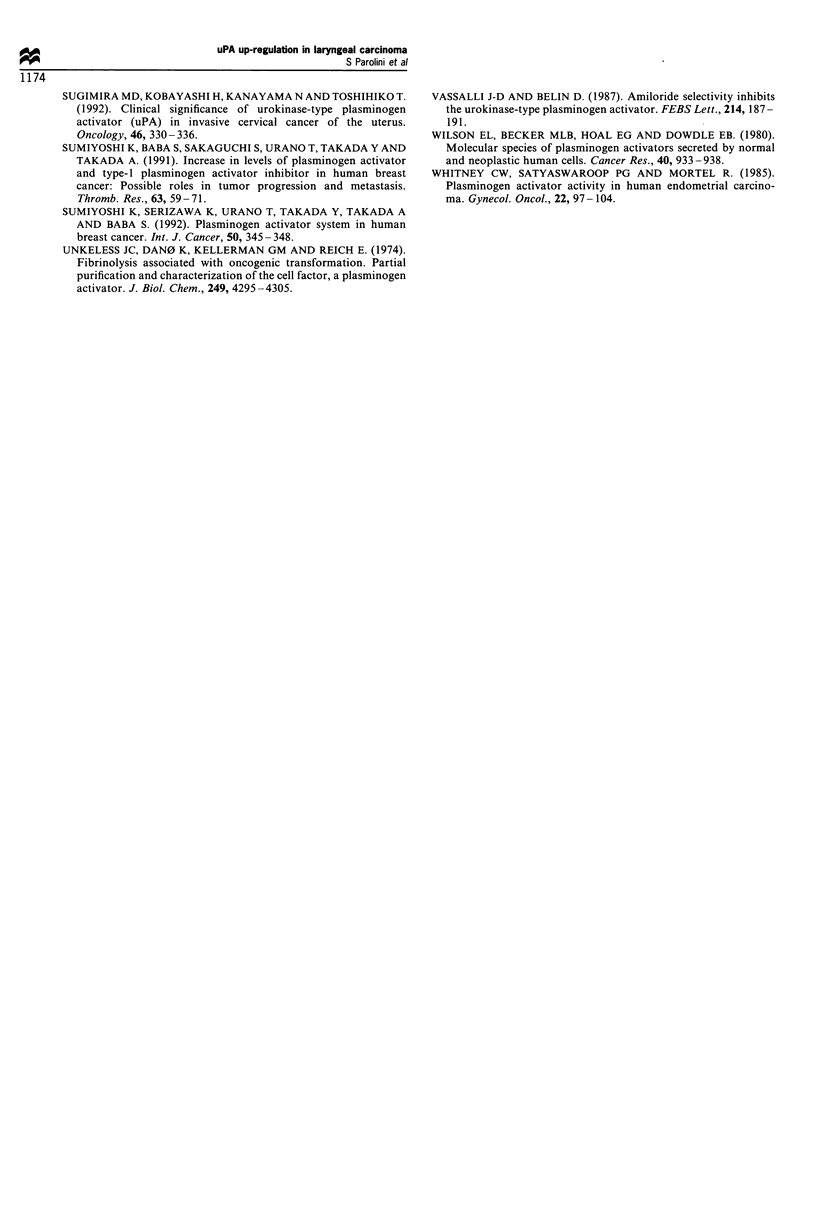# Up-regulation of urokinase-type plasminogen activator in squamous cell carcinoma of human larynx.

**DOI:** 10.1038/bjc.1996.512

**Published:** 1996-10

**Authors:** S. Parolini, D. Flagiello, A. Cinquetti, R. Gozzi, S. Cristini, J. Cappiello, P. Nicolai, M. Rusnati, M. Presta, M. M. Tosatti

**Affiliations:** Department of Biomedical Sciences and Biotechnology, University of Brescia, Italy.

## Abstract

**Images:**


British Journal of Cancer (1996) 74, 1168–1174
© 1996 Stockton Press All rights reserved 0007-0920/96 $12.00

### Up-regulation of urokinase-type plasminogen activator in squamous cell carcinoma of human larynx

S Parolini[1], D Flagiello[1], A Cinquetti[1], R Gozzi[1], S Cristini[1], J Cappiello[2], P Nicolai[2], M Rusnati[3], M Presta[3] and MP Molinari Tosatti[1]

[1]*Unit of Histology and Cytology, Department of Biomedical Sciences and Biotechnology;* [2]*Otorhinolaryngologic Clinic, School of Medicine and* [3]*Unit of Pathology and Immunology, Department of Biomedical Sciences and Biotechnology, University of Brescia, 25123 Brescia, Italy.*

**Summary** The expression of urokinase-type plasminogen activator (uPA) was investigated in squamous cell carcinoma of the human larynx. For this purpose, tissue extracts from 25 matched samples of normal mucosa and neoplastic larynx were compared for the levels of uPA activity as evaluated by a chromogenic PA assay and sodium dodecyl sulphate-polyacrylamide gel electrophoresis (SDS–PAGE) zymography. Also, uPA antigen was quantified by enzyme-linked immunosorbent assay (ELISA) in 19 cases. The results demonstrate a significant increase in the levels of uPA activity and protein in tumour tissue extracts, more pronounced in tumours with lymph node metastases. Immunohistochemistry performed on 70 biopsies showed that uPA positivity is present both in neoplastic cells and in fibroblast-like cells and macrophages. However, depending on the histological grading and invasive capacity of the tumour, a pronounced intra- and intertumoral heterogeneity in uPA staining was observed. *In situ* hybridisation confirmed the presence of uPA mRNA in both tumour and stromal cells. The present study provides experimental evidence for a role of uPA in the invasive growth of human laryngeal carcinoma.

**Keywords:** larynx carcinoma; urokinase; *in situ* hybridisation; immunohistochemistry; metastasis

Degradation of the extracellular matrix and other tissue barriers by proteases is a prerequisite for neoplastic growth and metastasis (Mignatti and Rifkin, 1993). Plasminogen activators (PAs) are serine-proteases which convert plasminogen to plasmin and regulate intravascular fibrinolysis and extracellular proteolysis. There are two types of PAs, tissue-type PA (tPA) and urokinase-type PA (uPA), which differ in molecular weight, immunological reactivity, enzymatic properties and genomic sequence (Danø *et al.*, 1985).

uPA is considered to play an important role in different processes where extracellular proteolysis is required, including cell migration, tissue remodelling during development, angiogenesis and invasive growth in normal and pathological conditions. In particular, several malignant tumours, e.g. carcinoma of the colon (Kohga *et al.*, 1985; De Bruin *et al.*, 1987*a*, *b*; Sim *et al.*, 1988; Nishino *et al.*, 1988; Pyke *et al.*, 1991), lung (Markus *et al.*, 1980; Sappino *et al.*, 1987), breast (Sumiyoshi *et al.*, 1992), uterus (Whitney *et al.*, 1985; Sugimira *et al.*, 1992), urinary bladder (Hasui *et al.*, 1989; 1992) and skin (Sappino *et al.*, 1991), express high levels of uPA compared with normal tissue. Numerous findings indicate that uPA is involved in tissue degradation during invasive growth by promoting breakdown of extracellular matrix proteins either by a direct action of plasmin or through a plasmin-mediated activation of latent collagenase (Ossowski *et al.*, 1983, 1988; Mignatti *et al.*, 1986; Saksela and Rifkin, 1988).

Previous observations had demonstrated a role for uPA in the *in vitro* invasive capacity of cell lines isolated from human squamous carcinoma of the oral cavity (Clayman *et al.*, 1993). Also, the determination of uPA antigen performed in the same study on a limited set of patients suggested a possible up-regulation of uPA expression in human laryngeal carcinomas *in situ*. On this basis, we decided to investigate more extensively the expression of uPA in the carcinoma of human larynx. In the present study, matched samples of normal mucosa and neoplastic laryngeal tissue were compared for levels of uPA activity and antigen, uPA localisation by immunohistochemistry, and uPA mRNA expression by *in situ* hybridisation. The findings demonstrate a significant increase in the levels of uPA activity and protein in the extracts of human laryngeal carcinoma, the extent of uPA up-regulation being related to the metastatic potential of the tumour. Accumulation of uPA antigen and mRNA was observed both in neoplastic and stromal cells. Our data point to a role for uPA in the invasive growth of human laryngeal carcinoma.

Correspondence: MP Molinari Tosatti, Unit of Histology and Cytology, Department Biomedical Sciences and Biotechnology, University of Brescia, via Valsabbina 19, 25123 Brescia, Italy
Received 11 September 1995; revised 23 April 1996; accepted 13 May 1996

### Materials and methods

### *Laryngeal biopsies*

Matched samples of normal mucosa and neoplastic laryngeal tissue were obtained from 70 patients who underwent total ($n=32$) or supraglottic ($n=38$) laryngectomy for laryngeal carcinoma. The normal mucosa sample was taken at least 3 cm from the neoplastic lesion. Samples showing large areas of necrosis were excluded from the study. Tissues were collected immediately after surgical removal and divided in two parts: one part was frozen at −70°C for the determination of uPA activity and antigen; the other one was fixed in 4% (w/v) paraformaldehyde for immunohistochemical studies and for *in situ* hybridisation. The histological diagnosis, performed on traditionally haematoxylin–eosin stained sections, identified the tumours as G1 ($n=15$), G2 ($n=30$) and G3 ($n=25$) squamous cell carcinomas according to the International Union Against Cancer-modified Broders' system and confirmed the absence of lesions in normal mucosa samples. Lymph node metastases were present in 30 cases. All 70 matched normal and tumour biopsies were examined for uPA immunostaining. Twenty-five G1, G2 and G3 matched samples (11 from metastatic tumours and 14 from non-metastatic tumours) were also examined for uPA levels by enzymatic activity assay and sodium dodecyl sulphate-polyacrylamide gel electrophoresis (SDS–PAGE) zymography. Nineteen out of the 25 samples were evaluated also by uPA enzyme-linked immunosorbent assay (ELISA) (7 from metastatic tumours and 12 from non-metastatic tumours). Finally, eight matched biopsies were analysed by *in situ* hybridisation.

*Preparation of tissue extracts and PA activity assay*

Frozen biopsies were thawed and homogenised in 1 ml of lysis buffer (0.05% Triton X-100/60 mM Tris-HCl, pH 8.5) per g of fresh tissue. Samples were centrifuged at $12\,000 \times g$ for 30 min at 4°C, and the supernatants were stored at −80°C until analysis. Protein concentration of the soluble extracts was determined by coomassie protein assay reagent (Pierce Europe, The Netherlands).

To evaluate the levels of tissue-associated PA activity, 30 μg of the tissue extracts were incubated in a 96-well microtitre plate with 42 nmol of the plasmin chromogenic substrate H-D-norleucyl-hexahydrotyrosil-lysine-p-nitroanilide-diacetate and 3 μg of purified human plasminogen (American Diagnostica, Greenwich, CT, USA) in 150 μl of lysis buffer (Presta *et al.*, 1989). After incubation at 37°C, the plate was read at 405 nm with an automatic microplate reader. A standard curve of human uPA (Calbiochem, La Jolla, CA, USA) was included in each assay.

*SDS–PAGE zymography and uPA ELISA*

For determination of the molecular weight of the PA activity in normal and neoplastic larynx, tissue extracts (700 μg of total protein) were run on SDS-10% polyacrylamide gel under non-reducing conditions. Then, proteins were electrophoretically transferred from the gel to a nitrocellulose membrane for 2 h at 400 mA in 40 mM sodium phosphate buffer, pH 6.5. Zymography of the proteins transferred to the membrane was carried out on a casein–agarose gel as described (Colombi *et al.*, 1986). Control gels were made in the absence of plasminogen to identify plasminogen-independent caseinolytic activities.

To evaluate the levels of uPA antigen, 50 μg of the tissue extracts were analysed by the IMUBIND uPA ELISA kit (American Diagnostica) according to manufacturer's instructions.

*Immunohistochemistry*

Specimens were fixed with 4% paraformaldehyde for 2 h at 4°C and cryoprotected through 5 h intervals of graded sucrose concentrations (5–30%) in phosphate-buffered saline (PBS). After embedding in OCT compound, samples were frozen, cut at 4 μm thickness using a freeze-microtome, and mounted on chrome–alum–gelatin-coated glass slides. After three rinses in 1% Triton X-100/PBS, sections were incubated for 30 min with 0.3% hydrogen peroxide in methanol to quench endogenous peroxidase activity. Slides were then washed three times for 10 min each in 1% Triton X-100/PBS, followed by a 30 min incubation at room temperature with 20% normal rabbit serum (Dakopatts, Denmark) in PBS to reduce non-specific background staining. After two rinses in PBS, sections were incubated overnight at 4°C with goat polyclonal anti-human uPA antibody (60 $\mu g\ ml^{-1}$ in PBS; Biopool, Sweden) or goat polyclonal anti-human tPA (12 $\mu g\ ml^{-1}$; Biopool), followed by 1 h incubation at room temperature. Sections were washed with PBS and incubated for 45 min with biotinylated rabbit anti-goat IgG (1:300) at room temperature. Formation of the antigen–antibody complexes was demonstrated with avidin–biotin–peroxidase complex (Dakopatts, Denmark), and bound peroxidase was developed with 3-amino-9-ethylcarbazole (Sigma) in acetate buffer, pH 5.2, added with hydrogen peroxide. The slides were lightly counterstained with haematoxylin and mounted.

The specificity of immunostaining was verified by: (1) omission of the first, second or third layer of antiserum; (2) substitution of the primary antibody with non-immune IgG; and (3) absorption of the primary antibody with highly purified antigen coupled to Sepharose beads. The results obtained with the polyclonal anti-uPA antibody were confirmed in 20 cases by staining of adjacent sections with a monoclonal anti-uPA antibody (Ab no. 3689, American Diagnostica).

In situ *hybridisation*

The probe used was a 1.3 kb *SmaI*/*Bam*HI fragment obtained from the pcUK176 recombinant plasmid harbouring the human uPA cDNA (provided by P Mignatti, University of Pavia). The probe (500 ng) was labelled to a specific activity of $1.5 \times 10^7$ c.p.m. $\mu g^{-1}$ by using the random primed DNA labelling method according to supplier's instructions (Molecular Biology, Boehringer Mannheim) in the presence of 6 μM [$^3$H]dATP (92 Ci $mM^{-1}$) (Amersham, Buckinghamshire, UK) and 6 μM each of unlabelled dCTP, dGTP and dTTP. The probe was separated from unincorporated nucleotides by precipitation with ethanol, dried and dissolved in the hybridisation buffer.

Routinely processed, paraformaldehyde-fixed sections were subjected to *in situ* hybridisation according to Moro *et al.* (1992). Briefly, slides were sequentially immersed twice in PBS for 10 min, once in 1 $\mu g\ ml^{-1}$ proteinase K dissolved in 2 mM calcium chloride/10 mM Tris-HCl, pH 7.4, for 8 min at 37°C, and twice in 2 $\mu g\ ml^{-1}$ glycine in PBS for 3 min. Sections were then acetylated in freshly prepared 0.25% acetic anhydride in 0.1 M triethanolamine-HCl, pH 8.0, for 10 min (Hayashi *et al.*, 1978). Finally the slides were washed twice in PBS for 3 min, dehydrated in 50%, 75%, 95% and 100% ethanol, 5 min for each passage, and air-dried. The hybridisation was performed in a mixture containing 1 $\mu g\ ml^{-1}$ $^3$H-labelled probe prepared according to Moro *et al.* (1992), heated at 70°C for 10 min and cooled in ice. Aliquots (30 μl) were applied onto each section and incubated at 42°C for 16–18 h. Slides were rinsed four times in 50% formamide/ 2 × SSC for 15 min at 39°C and twice in 1 × SSC for 30 min at room temperature. Autoradiography was performed by dipping the slides into Kodak NTB2 emulsion, air-drying for 2 h in the dark, and storing for 4 weeks in sealed boxes at 4°C. Slides were developed in Kodak D19 developer, fixed in Kodak fixer, rinsed in water, air-dried, stained with haematoxylin–eosin and mounted with glycerol–gelatin. For each specimen, controls included incubation with pBR322-labelled probe or with hybridisation mix only.

*Image anslysis*

The Magiscan image analysis system (Joyce Loebl, Gateshead, UK) was used to perform semi-quantitative evaluation of the hybridisation signals, according to Moro *et al.* (1992). Briefly, the section image was input via a TV camera mounted on a Nikon light microscope, digitalised on the high resolution monitor and stored within the Magiscan's image memory. Five fields were chosen at random and analysed per slide. The ratio between the integrated density values of the area of the hybridisation grains and of the selected surface was determined for each field and non-specific hybridisation signals were subtracted from all values. This procedure has been used previously to quantify gene expression both in cultured cells and in histological sections (Moro *et al.*, 1990, 1992; Colombi *et al.*, 1991, 1993).

*Statistical analysis*

Student's *t*-test was used for comparison of the levels of PA activity, uPA antigen or uPA hybridisation signals among normal and tumour samples. Values are expressed as mean ± s.e.m.

### Results

*uPA levels in laryngeal tumours*

To evaluate the modifications of uPA activity that follow the transformation of human larynx, matched samples of normal and tumoral laryngeal tissues were analysed in 25 out of the 70 patients examined. PA activity was determined in tissue extracts with a plasmin chromogenic assay and expressed as

**Figure 1** uPA in human laryngeal tumours. Paired samples of normal (N) and tumoral (T) laryngeal tissue extracts were evaluated for uPA activity with a chromogenic assay (**a**) and for the levels of uPA antigen by ELISA (**b**). Patients with non-metastatic tumours were compared with patients bearing tumours with lymph node involvement.

Committee on Thrombolytic Agents (CTA) Units mg$^{-1}$ of protein (Figure 1a). Preliminary experiments had indicated that PA activity of laryngeal tissue extracts was abolished by preincubation of the samples with 1 mM amiloride (Vassalli and Belin, 1987), thus confirming its identity with uPA activity (data not shown). uPA activity was higher in the extracts of laryngeal carcinomas than in matched normal tissues. It should be noted that uPA activity was equal or below the limits of detection of the assay (0.1 U mg$^{-1}$ of protein) in 16 normal tissue extracts but only in two tumour extracts (for statistical analysis uPA activity in these samples was assumed to be equal to 0.1 U mg$^{-1}$ of protein). The mean value$\pm$s.e.m. of uPA activity in the cancer group was $0.88 \pm 0.21$ U mg$^{-1}$ of protein, five times higher than in the normal group ($0.17 \pm 0.03$ U mg$^{-1}$ of protein; $P<0.01$). Interestingly, the levels of uPA activity were significantly higher in the extracts of primary cancers with positive lymph node involvement ($n=11$) than in those of non-metastatic tumours ($n=14$) ($1.35 \pm 0.42$ *vs* $0.51 \pm 0.12$ U mg$^{-1}$ of protein respectively; $P<0.05$). No significant differences in uPA activity levels were observed when tumours were compared according to their histological grading ($0.42 \pm 0.20$, $1.00 \pm 0.32$ and $0.86 \pm 0.19$ U mg$^{-1}$ of protein for G1, G2 and G3 tumours respectively).

Analysis of tissue extracts by SDS-PAGE, combined with detection of PA activity by zymographic assay on casein–agarose gel, was used to confirm the identity of the type of PA produced by normal and neoplastic larynx. Purified human uPA and tPA were used as standards. As shown in Figure 2, zymography of normal and tumoral laryngeal tissue extracts revealed a major lytic band with an apparent molecular weight of 53 000, comigrating with the uPA standard. The band was not apparent when plasminogen was omitted from the casein–agarose gel (data not shown), thus confirming its identity with uPA. No lytic bands comigrating with the tPA standard were observed in normal or cancer tissue extracts. In agreement with the chromogenic PA activity assay, SDS-PAGE zymography confirmed that uPA activity was significantly higher in tumoral than in matched normal tissue extracts for all the patients examined and that the diagnosis of positive lymph node involvement was characterised by the highest levels of uPA activity (Figure 2).

To assess whether the increased levels of uPA activity detected in tumour samples reflected an increase in uPA antigen content, an ELISA was used on 19 out of the 25 matched biopsies analysed above. As shown in Figure 1b, the

**Figure 2** Casein–agarose zymography of laryngeal tumour extracts. Tissue extracts (700 $\mu$g of protein) were run on SDS–10% polyacrylamide gel. Zymography of the proteins transferred to nitrocellulose membrane was carried out on a casein–agarose gel containing human plasminogen. The migration of purified human uPA and tPA run on a parallel gel is shown by arrows. No lytic bands were observed when plasminogen was omitted from the gel. N, normal larynx; T, non-metastatic tumour; MT, metastatic tumour.

levels of uPA antigen were higher in the extracts of laryngeal carcinomas than in matched normal tissues ($0.27 \pm 0.05$ *vs* $1.72 \pm 0.31$ ng uPA per mg of protein for control and tumour biopsies respectively; $P < 0.01$). It must be pointed out that a linear relationship was found when the levels of uPA antigen measured in each tumour biopsy were plotted against the corresponding levels of uPA activity ($r = 0.87$, $P < 0.01$). Also, in agreement with the uPA activity data, the levels of uPA antigen were significantly higher in primary cancers with positive lymph node involvement ($n = 7$) than in non-metastatic tumours ($n = 12$) ($2.62 \pm 0.44$ *vs* $1.19 \pm 0.35$ ng uPA per mg of protein respectively; $P < 0.02$), while no significant differences were observed among biopsies from tumours with different histological grading (data not shown).

In conclusion, our data indicate that the transformation of normal laryngeal mucosa to neoplastic tissue is associated with an increase in tissue levels of uPA. This increase appears to be related to the metastatic potential of the tumour but not to its histological grading.

### *Immunolocalisation of uPA*

The immunostaining pattern with polyclonal anti-uPA antibody was evaluated in cryostat sections from all 70 biopsies of normal larynx and paired laryngeal squamous cell carcinomas. Staining controls included deletion of the various antibody layers, the use of non-immune IgG, and the use of antibody preparations absorbed with highly purified preparations of the corresponding antigen coupled to Sepharose beads. The controls were negative in all cases. In selected cases, uPA immunostaining was confirmed by using a monoclonal anti-uPA antibody (see Materials and methods).

In normal laryngeal mucosa, a weak staining for uPA was found in epithelium and in a few fibroblast-like cells and macrophages (not shown). In laryngeal carcinoma, uPA immunoreactivity was distributed heterogeneously both in metastatic and non-metastatic tumours (Figure 3a–d). In detail, we observed that in 12 out of the 15 G1 tumours examined most of the cellular nests were positive for uPA staining (Figure 3a), while in remaining G1 tumours and in all G2 tumours examined cellular nests characterised by a strong uPA staining and areas completely devoid of uPA immunoreactivity were present within the same biopsy (Figure 3b). Interestingly, in these cases the most intense reaction was usually detectable in tumour cells that appeared to be well differentiated according to histological criteria, whereas positivity decreased in poorly differentiated cells (Figure 3b). IN G3 tumours, uPA positivity was mainly localised in areas with invasive growth and degradation of surrounding tissue (Figure 3c). Despite this heterogeneity, a general feature of the larynx tumours examined was that primary tumours with lymph node involvement were characterised by a more intense uPA staining in the cytoplasm of tumour cells than that observed in non-metastatic tumours. Also, stromal fibroblast-like cells and macrophages showed uPA positivity in all tumours independently of the histological grading and lymph node involvement (Figure 3d).

**Figure 3** Immunohistochemical localisation of uPA in human laryngeal tumours. Cryostat sections were stained with polyclonal anti-uPA antibody as described in Materials and methods. (**a**) Highly differentiated tumour in which all cells within the nests show an intense cytoplasmic uPA staining (original magnification: 10 ×). (**b**) Moderately differentiated tumour in which uPA is detectable only in the most differentiated cells (original magnification: 25 ×). (**c**) uPA staining associated with the invasive front of a G3 tumour (arrows, original magnification: 16 ×). (**d**) Numerous uPA-positive cells (arrows) in peritumoral stroma with inflammatory infiltrate (original magnification: 25 ×).

**Figure 4** Localisation of uPA mRNA in human laryngeal tumours by *in situ* hybridisation. Hybridisation was performed on cryostat sections by using a $^{3}$H-labelled uPA cDNA probe. Low expression of uPA mRNA is detectable in normal epithelium (**b**). Higher levels of uPA mRNA expression are present in tumour cells (**c**). Specificity of the hybridisation is evident from the absence of hybridisation in slides incubated with control pBR322 probe (**a**). Original magnification: 63 ×.

When samples were analysed for tPA immunoreactivity, positivity was limited to capillary endothelial cells in both normal and malignant tissue. No tPA staining was located in neoplastic cells (data not shown).

*uPA mRNA expression in laryngeal cancer using* in situ *hybridisation*

To identify the cell types responsible for uPA production in laryngeal carcinoma, *in situ* hybridisation with a specific human uPA probe was performed in eight cases of normal larynx and corresponding laryngeal carcinoma of different histological grading. In all cases examined, neoplastic cells expressed uPA mRNA (Figure 4). Hybridisation grains were also observed in fibroblast-like cells and macrophages in the tumoral stroma, whereas endothelial cells were negative (data not shown). Semi-quantitative evaluation of uPA mRNA levels was attempted by computerised image analysis of the *in situ* hybridisation signals according to Moro *et al.* (1992). The results demonstrate a significant increase in uPA mRNA expression in tumour samples (integrated density per unit area equal to $5.2 \pm 0.8$ *vs* $1.5 \pm 0.2$ in tumour *vs* normal samples, $P < 0.01$). These data, even though obtained on a limited number of cases and with a semi-quantitative method, suggest that the increased levels of uPA antigen detected in tumour samples depend, at least in part, on an increased expression of uPA gene. Further experiments are necessary to confirm this hypothesis.

### Discussion

Our findings demonstrate that uPA is up-regulated in the squamous cell carcinoma of human larynx. Increased levels of uPA activity and protein were detectable in the extracts of all tumours examined when compared with normal tissue obtained from the same patients. uPA immunostaining was present in tumour cells as well as in macrophages and fibroblast-like cells of the tumour stroma. Accordingly, *in situ* hybridisation showed uPA gene expression to occur both in parenchymal and stromal cells. At variance with uPA, no modifications in activity levels or immunostaining were observed for tPA in laryngeal carcinoma. tPA remains confined to the endothelium and tumour cells do not express it. Thus, the increased fibrinolytic potential observed in laryngeal carcinomas is a result of uPA up-regulation. This is in keeping with the general observation that tumour cells produce mainly uPA, with few exceptions represented by melanoma, neuroblastoma and certain leukaemia cells (Wilson *et al.*, 1980; Rijken and Collen, 1981; Neuman *et al.*, 1989).

It was demonstrated in the early 1970s that the transformation of cultured cells by oncogenic viruses caused a significant increase in the production of uPA (Ossowski *et al.*, 1973; Goldberg, 1974; Unkeless *et al.*, 1974). Since then, several observations *in vitro* and *in vivo* have linked the expression of uPA to the transformed state. For instance, tumour tissue extracts from carcinomas of the colon, breast, lung and bladder all contained more uPA antigen and/or activity than did their normal counterparts (Markus *et al.*, 1980; Corasanti *et al.*, 1980; Camiolo *et al.*, 1981, 1984). Interestingly, different immunohistochemical studies have shown that uPA is localised in invading areas of the tumour, supporting the hypothesis of a role for uPA in tumour cell invasion (Skriver *et al.*, 1984; Kohga *et al.*, 1985; Kristensen *et al.*, 1990). Indeed, neutralising anti-uPA antibodies have been demonstrated to inhibit tumour cell invasion in different experimental models *in vitro* and *in vivo* (Ossowski and Reich, 1983; Hearing *et al.*, 1988). Also, a positive relationship between uPA up-regulation in primary tumour and the presence of lymph node metastasis has been demonstrated for different neoplasms, including lung cancer (Sappino *et al.*, 1987), breast carcinoma (Sumiyoshi *et al.*, 1991) and cervical cancer of the uterus (Sugimira *et al.*, 1992). Here, we have shown that levels of uPA activity and uPA antigen in tumour extracts were significantly higher in laryngeal tumours with lymph node metastasis than in non-metastatic tumours, suggesting a possible correlation among uPA expression, invasiveness and metastatic potential in human laryngeal carcinoma. Indeed, we have observed uPA immunoreactivity in invasive areas of G3 laryngeal tumours. These observations are in keeping with previous findings on the role of uPA in mediating the capacity of cultured squamous cell carcinoma cell lines to invade a reconstituted extracellular matrix (Matrigel) in an *in vitro* assay (Clayman *et al.*, 1993). Thus, our results further support a positive correlation between uPA production, infiltrative growth and metastatic ability of human tumours, indicating that uPA up-regulation may be considered a parameter of malignancy.

Immunohistochemical localisation of uPA in laryngeal carcinomas demonstrated intratumoral heterogeneity in the cellular distribution of the enzyme, more evident in G2 and G3 tumours. Also, heterogeneous expression of uPA gene was observed by *in situ* hybridisation among tumour cells within the same neoplasm (see Figure 4c). This may reflect the heterogeneity in cell population typical for most malignant neoplasms (Skriver *et al.*, 1984; Kristensen *et al.*, 1990) and may be related to focal dissolution of the basement membrane observed in these tumours (Antonelli *et al.*, 1991). In apparent contrast with these observations is the more homogeneous distribution of uPA positivity in cell nests of G1 tumours. Since the antibodies used in the present study do not discriminate between active uPA and inactive proenzyme, it is possible that uPA is present in G1 tumours as inactive pro-uPA that is rapidly converted to the active form by trace amounts of plasmin during the chromogenic and SDS-PAGE zymography assays. Also, modifications of the levels of PA inhibitors may contribute to the final proteolytic balance of laryngeal carcinomas. Further experiments are required to clarify this point.

Our data demonstrate that parenchymal cells and stromal cells within the laryngeal tumour produce uPA, thus suggesting that a complex interplay may exist between both cell types in generating a profibrinolytic environment in human laryngeal cancer. uPA gene expression in cultured oral cavity squamous cell carcinoma cell lines has been reported previously (Clayman *et al.*, 1993). Also, malignant cells of cutaneous squamous cell carcinoma express uPA *in situ* (Sappino *et al.*, 1991). In contrast, previous studies had shown that the presence of uPA mRNA in human colon adenocarcinomas is limited to stromal cells adjacent to invasive nodules, while tumour cells express uPA-receptor gene only (Pyke *et al.*, 1991). Taken together, these observations suggest that the cell localisation of uPA gene expression can vary among different tumour types and that tumour cells and/or stromal cells may contribute to the fibrinolytic balance in human neoplasms.

In conclusion, the present study provides experimental evidence for a role of uPA in the invasive growth of squamous cell laryngeal carcinoma. The determination of uPA antigen in breast cancer tissue extracts has been shown to represent a significant prognostic factor for disease-free interval and total survival (Janicke *et al.*, 1990, 1992; Schmitt *et al.*, 1990; Grøndahl-Hansen *et al.*, 1993). Further studies are required to assess whether uPA up-regulation also represents a useful prognostic factor for laryngeal carcinoma.

**Acknowledgements**
This work was supported in part by grants from CNR (no. 94.00710.CT11) and MURST (40%) to MP Molinari Tosatti and from MURST (60%), CNR (no. 94.00316.CT14) and Associazione Italiana per la Ricerca sul Cancro to M Presta.

### References

ANTONELLI AR. NICOLAI P. CAPPIELLO J. PERETTI G. MOLINARI TOSATTI MP. ROSA D. GRIGOLATO PG. FAVRET M AND MAROCCOLO D. (1991). Basement membrane components in normal. dysplastic. neoplastic laryngeal tissue and metastatic lymph nodes. *Acta Otolaryngol.* **111,** 437-443.

CAMIOLO SM. MARKUS G. EVERS JL. HOBIKA GH. DEPASQUALE JL. BECKLEY S AND GRIMALDI JP. (1981). Plasminogen activator content of neoplastic and benign human prostate tissues: fibrin augmentation of an activator activity. *Int. J. Cancer.* **27,** 191-198.

CAMIOLO SM. MARKUS G. ENGLANDER LS. SIUTA MR. HOBIKA GH AND KOHGA S. (1984). Plasminogen activator content and secretion in explants of neoplastic and benign human prostate tissues. *Cancer Res.*. **44,** 311-318.

CLAYMAN G. WANG SW. NICOLSON GL. EL-NAGGAR A. MAZAR A. HENKIN J. BLASI F. GOEPFERT H AND BOYD DD. (1993). Regulation of urokinase-type plasminogen activator expression in squamous-cell carcinoma of the oral cavity. *Int. J. Cancer.* **54,** 73-80.

COLOMBI M. BARLATI S. MAGDELENAT H AND FISZER-SZAGARZ B. (1986). Relationship between multiple forms of plasminogen activator in human breast tumors and plasma and presence of metastasis in lymph nodes. *Cancer Res.*. **44,** 2971-2975.

COLOMBI M. MORO L. ZOPPI N. GHINELLI A AND BARLATI S. (1991). Altered fibronectin mRNA splicing in skin fibroblast from Ehlers-Danlos syndrome patients: *in situ* hybridization analysis. *Cell Biol. Int. Rep.*. **15,** 1195-1205.

COLOMBI M. MORO L. ZOPPI N AND BARLATI S. (1993). Quantitative evaluation of mRNAs by *in situ* hybridation and image analysis: principles and applications. *DNA Cell Biol.*. **12,** 629-636.

CORASANTI JG. CELIK C. CAMIOLO SM. MITTELMAN A. EVERS JL. BARBASCH A. HOBLKA GH AND MARKUS G. (1980). Plasminogen activator content of human colon tumors and normal mucosae: Separation of enzymes and partial purification. *J. Natl Cancer Inst.*. **65,** 345-351.

DANØ K. ANDREASEN PA. GRØNDAHL-HANSEN J. KRINSTENSEN JP. NIELSEN LS AND SKRIVER L. (1985). Plasminogen activators. tissue degradation and cancer. *Adv. Cancer Res.*. **44,** 139-266.

DE BRUIN PAF. VERSPAGET HW. GRIFFERON G. NAP M. VERHEIJEN JH AND LAMERS CBHW. (1987*a*). Plasminogen activator activity and composition in human colorectal carcinomas. *Fibrinolysis.* **1,** 57-62.

DE BRUIN PAF. GRIFFERON G. VERSPAGET HW. VERHEIJEN JH AND LAMERS CBHW. (1987*b*). Plasminogen activators and tumour development in the human colon: activity levels in normal mucosa. adenomatous polyps and adenocarcinomas. *Cancer Res.*. **47,** 4654-4657.

GOLDBERG AR. (1974). Increased protease levels in transformed cells: a casein overlay for the detection of plasminogen activator production. *Cell.* **2,** 95-102.

GRØNDAHL-HANSEN J. CHRISTENSEN IJ. ROSENQUIST C. BRUNNER N. MOURIDSEN HT. DANØ K AND BLICHERT-TOFT M. (1993). High levels of urokinase-type plasminogen activator and its inhibitor PAI-1 in cytosolic extracts of breast carcinomas are associated with poor prognosis. *Cancer Res.*. **53,** 2513-2521.

HASUI Y. SUZUMINYA J. MARUTSUKA K. SUMIYOSHI A. HASHIDA S AND ISHIKHAWA EI. (1989). Comparative study of plasminogen activators in cancers and normal mucosae of human urinary bladder. *Cancer Res.*. **49,** 1067-1070.

HASUI Y. MARUTSUKA K. SUZUMIYA J. KITAOTA S. OSADA Y AND SUMIYOSHI A. (1992). The content of urokinase-type plasminogen activator antigen as a prognostic factor in urinary bladder cancer. *Int. J. Cancer.* **50,** 871-873.

HAYASHI S. GILLAM IC. DELANEU AD AND TENER GM. (1978). Acetylation of chromosome squashes of *Drosophila melanogaster* decreases the background in autoradiographs from hybridization with $^{125}$I-labeled RNA. *J. Histochem. Cytochem.*. **26,** 677-679.

HEARING JV. LAW LW. CORTI A. APPELLA E AND BLASI F. (1988). Modulation of metastatic potential by cell surface urokinase of murine melanoma cells. *Cancer Res.*. **48,** 1270-1278.

JANICKE F. ACHMITT M. HAFTER R. HOLLRIEDER A. BABIC R. ULM K. GOSSNER W AND GRAEFF H. (1990). Urokinase-type plasminogen activator (uPA) antigen is a predictor of early relapse in breast cancer. *Fibrinolysis.* **4,** 69-78.

JANICKE E. SCHMITT M. MOUIWA M. CHUCHOLOWSKI N. PACHE L AND GRAEFF M. (1992). Tumor-associated urokinase-type plasminogen activator: biological and clinical significance. *Biol. Chem. Hoppe-Seyler.* **373,** 611-622.

KOHGA S. HARVEY S AND MARKUS G. (1985). Localization of plasminogen activators in human colon cancer by immunoperoxidase staining. *Cancer Res.*. **45,** 1787-1796.

KRISTENSEN P. PYKE C. LUND LR. ANDREASEN PA AND DANØ K. (1990). Plasminogen activator inhibitor-type 1 in Lewis lung carcinoma. *Histochemistry.* **93,** 559-566.

MARKUS G. TAKITA II. CAMIOLO SM. CORASANTI JG. EVERS JI AND HOBIKA GH. (1980). Content and characterization of plasminogen activators in human lung tumors and normal lung tissue. *Cancer Res.*. **40,** 841-848.

MIGNATTI P. ROBBINS E AND RIFKIN DB. (1986). Tumor invasion through the human amniotic membrane: requirement for a proteinase cascade. *Cell.* **47,** 487-498.

MIGNATTI P AND RIFKIN DB. (1993). Biology and biochemistry of proteinases in tumor invasion. *Physiol. Rev.*. **73,** 161-195.

MORO L. COLOMBI M. CRESPI S. DI LERNIA R AND BARLATI S. (1990). Study of fibronectin expression in tumour cells by dot-blot and *in situ* hybridization: quantitative evaluation by image analysis. *Cell Biol. Int. Rep.*. **14,** 701-715.

MORO L. COLOMBI M. MOLINARI TOSATTI MP AND BARLATI S. (1992). Study of fibronectin mRNA in human laryngeal and ectocervical carcinomas by *in situ* hybridization and image analysis. *Int. J. Cancer.* **51,** 692-697.

NEUMAN T. STEPHENS RW. SALONEN EM. TIMMUSK T AND VAHERI A. (1989). Induction of morphological differentiation of human neuroblastoma cells is accompanied by induction of tissue-type plasminogen activator. *J. Neurosci. Res.*. **23,** 274-281.

NISHINO N. AOKI K. TOKURA Y. SAKAGUCHI S. TAKADA Y AND TAKADA A. (1988). The urokinase type of plasminogen activator in cancer of digestive tracts. *Thromb. Res.*. **50,** 527-535.

OSSOWSKI L. (1988). *In vivo* invasion of modified chorioallantoic membrane by tumor cells: the role of cell surface-bound urokinase. *J. Cell Biol.*. **107,** 2437-2445.

OSSOWSKI L AND REICH E. (1993). Antibodies to plasminogen activator inhibit human tumor metastasis. *Cell.* **35,** 611-619.

OSSOWSKI L. UNKELESS JC. TOBIA A. QUIGLEY JP. RIFKIN DB AND REICH E. (1973). An enzymatic function associated with transformation of fibroblasts by oncogenic viruses. II Mammalian fibroblast cultures transformed by DNA and RNA tumor viruses. *J. Exp. Med.*. **137,** 112-126.

PRESTA M. MAIER JAM AND RAGNOTTI G. (1989). The mitogenic signaling pathway but not the plasminogen activator-induced pathway of basic fibroblast growth factor is mediated through protein kinase C in fetal bovine aortic endothelial cells. *J. Cell Biol.*. **109,** 1877-1884.

PYKE C. KRISTENSEN P. RALFKIAER E. GRØNDAL-HANSEN J. ERIKSEN J. BLASI F AND DANO H. (1991). Urokinase-type plasminogen activator is expressed in stromal cells and its receptor in cancer cells at invasive foci in human colon adenocarcinomas. *Am. J. Pathol.*. **138,** 1059-1067.

RIJKEN DC AND COLLEN D. (1981). Purification and characterization of the plasminogen activator secreted by human melanoma cells in culture. *J. Biol. Chem.*. **256,** 7053-7041.

SAKSELA O AND RIFKIN DB. (1988). Cell-associated plasminogen activation: regulation and physiological functions. *Annu. Rev. Cell Biol.*. **4,** 93-126.

SAPPINO A-P. BUSSO N. BELIN D AND VASSALLI J-D. (1987). Increase of urokinase-type plasminogen activator gene expression in human lung and breast carcinomas. *Cancer Res.*. **47,** 4043-4046.

SAPPINO A-P. BELIN D. HUARTE J. HIRSCHEL-SCHOLZ S. SAURAT J-H AND VASSALLI J-D. (1991). Differential protease expression by cutaneous squamous and basal cell carcinomas. *J. Clin. Invest.*. **88,** 1073-1079.

SCHMITT M. JANICKE F AND GRAEFF H. (1990). Tumor-associated fibrinolysis: the prognostic relevance of plasminogen activators uPA and tPA in human breast cancer. *Blood Coagul. Fibrinol.*. **1,** 695-702.

SIM P-S. STEPHENS RW. FAYLE DRH AND DOE WF. (1988). Urokinase-type plasminogen activator in colorectal carcinomas and adenomatous polyps: quantitative expression of active and proenzyme. *Int. J. Cancer.* **42,** 483-488.

SKRIVER L. LARSSONN LI. KIELBERG V. NIELSEN LS. ANDERSONS PB. KRISTENSENS P AND DANØ K. (1984). Immunocytochemical localization of urokinase-type plasminogen activator in Lewis lung carcinoma. *J. Cell. Biol.*. **99,** 752-757.

SUGIMIRA MD, KOBAYASHI H, KANAYAMA N AND TOSHIHIKO T. (1992). Clinical significance of urokinase-type plasminogen activator (uPA) in invasive cervical cancer of the uterus. *Oncology*, **46**, 330–336.

SUMIYOSHI K, BABA S, SAKAGUCHI S, URANO T, TAKADA Y AND TAKADA A. (1991). Increase in levels of plasminogen activator and type-1 plasminogen activator inhibitor in human breast cancer: Possible roles in tumor progression and metastasis. *Thromb. Res.*, **63**, 59–71.

SUMIYOSHI K, SERIZAWA K, URANO T, TAKADA Y, TAKADA A AND BABA S. (1992). Plasminogen activator system in human breast cancer. *Int. J. Cancer*, **50**, 345–348.

UNKELESS JC, DANØ K, KELLERMAN GM AND REICH E. (1974). Fibrinolysis associated with oncogenic transformation. Partial purification and characterization of the cell factor, a plasminogen activator. *J. Biol. Chem.*, **249**, 4295–4305.

VASSALLI J-D AND BELIN D. (1987). Amiloride selectivity inhibits the urokinase-type plasminogen activator. *FEBS Lett.*, **214**, 187–191.

WILSON EL, BECKER MLB, HOAL EG AND DOWDLE EB. (1980). Molecular species of plasminogen activators secreted by normal and neoplastic human cells. *Cancer Res.*, **40**, 933–938.

WHITNEY CW, SATYASWAROOP PG AND MORTEL R. (1985). Plasminogen activator activity in human endometrial carcinoma. *Gynecol. Oncol.*, **22**, 97–104.